# Commentary: If it looks too good to be true, it probably is

**DOI:** 10.1016/j.xjtc.2021.06.036

**Published:** 2021-07-01

**Authors:** Frank W. Sellke

**Affiliations:** Division of Cardiothoracic Surgery, Lifespan Cardiovascular Institute, Alpert Medical School of Brown University, and Rhode Island Hospital, Providence, RI

**Figure:**
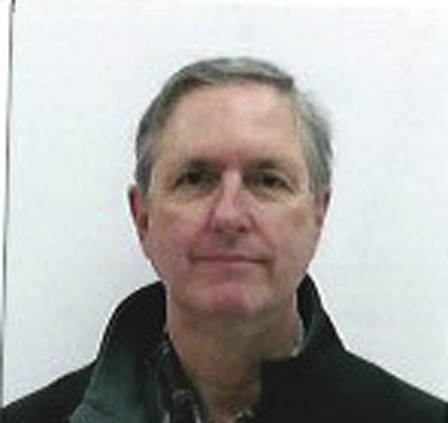
Frank W. Sellke, MD

**Central Message**

NLRP3 inhibition improved heart function in a rodent model of circulatory death donation, but most findings in rodent models lead to no real benefit when examined in human patients.

See Article page 89.

Heart transplantation is markedly limited by a lack of availability of donor hearts. Preliminary trials have shown that the use of hearts donated after circulatory death (DCD) is feasible and 1 manner in which to potentially increase the number of hearts available for transplantation. However, there are biologic ad logistical issues related to hearts and other organs DCD. Strategies to reduce ischemia/reperfusion injury in DCD hearts is 1 way to potentially facilitate the routine use of these hearts for transplantation. This brief research communication^1^ investigates the effects of the NLR family pyrin domain containing 3 (NLRP3) inflammasome, a mediator of myocardial ischemic injury and remodeling. The effects of NLRP3 inhibition on heart function was examined in a mouse model of circulatory death donor in a model of transplantation. The study is appropriate in that 1 group was given an NLRP3 inhibitor and compared with a group not given the inhibitor. The authors further demonstrated that the apoptosis-associated speck-like protein containing a caspase recruiting domain was increased in DCD control hearts but significantly less so in the DCD NLRP-I group hearts. The improvement is striking, and if these studies translate into real clinical improvement in donor heart numbers, the authors should book their tickets for Stockholm to pick up their Nobel Prize. However, many things, in fact most things, work in rodents and even larger animal models but do not work in human patients.^2^ Indeed, most preliminary findings in rodent models have not translated into any clinical benefit. Performing these studies in a larger animal model would provide more clinically relevant information, although there is no assurance of this. Even studies that have demonstrated physiologic findings in large animal models or even patients does not necessarily lead to improved outcomes in human patients. Indeed, our own work studying the effects of cardioplegia type on myocardial and vascular protection^3^, ^4^, ^5^ has found improvements in the large animal models but has not translated into a real improvement in human patient care.^6^ In this study,^1^ a better assessment of histologic, inflammatory, and biochemical changes would have provided more supportive and mechanistic information. Having stated that, this study provides compelling data to support the use of NLRP3 inhibition and other adjuvant methods to improve heart function in the setting of DCD hearts. The only way to find if there is an actual benefit of NLRP3 inhibition is to examine it in a clinical trial.
